# Sex-Related Differences in Reproductive Allocation, Growth, Defense and Herbivory in Three Dioecious Neotropical Palms

**DOI:** 10.1371/journal.pone.0009824

**Published:** 2010-03-23

**Authors:** Verónica Cepeda-Cornejo, Rodolfo Dirzo

**Affiliations:** 1 Departamento de Ecología Evolutiva, Instituto de Ecología, Universidad Nacional Autónoma de México, México, Distrito Federal, México; 2 Department of Biology, Stanford University, Stanford, California, United States of America; Institut Mediterrani d'Estudis Avançats (CSIC/UIB), Spain

## Abstract

**Background:**

Frequently, in dioecious plants, female plants allocate more resources to reproduction than male plants. Therefore it is expected that asymmetrical allocation to reproduction may lead to a reproduction-growth tradeoff, whereby female plants grow less than male plants, but invest more in defenses and thus experience lower herbivory than male plants.

**Methodology/Principal Findings:**

We tested these expectations by comparing resource allocation to reproduction, growth and defense and its consequences on herbivory in three sympatric dioecious *Chamaedorea* palms (*C. alternans*, *C. pinnatifrons* and *C. ernesti-augusti*) using a pair-wise design (replicated male/female neighboring plants) in a Mexican tropical rain forest. Our findings support the predictions. Biomass allocation to reproduction in *C. pinnatifrons* was 3-times higher in female than male plants, consistent with what is known in *C. alternans* and *C. ernesti-augusti*. Growth (height and leaf production rate and biomass production) was higher in male plants of all three species. Female plants of the three species had traits that suggest greater investment in defense, as they had 4–16% tougher leaves, and 8–18% higher total phenolic compounds concentration. Accordingly, female plants sustained 53–78% lower standing herbivory and 49–87% lower herbivory rates than male plants.

**Conclusions/Significance:**

Our results suggests that resource allocation to reproduction in the studied palms is more costly to female plants and this leads to predictable intersexual differences in growth, defense and herbivory. We conclude that resource allocation to reproduction in plants can have important consequences that influence their interaction with herbivores. Since herbivory is recognized as an important selective force in plants, these results are of significance to our understanding of plant defense evolution.

## Introduction

The Resource Allocation Principle states that if resources are limited, two or more functions compete directly and an increase in resources allocated to one function will result in a decrease in resources allocated to the other(s) [1]. In general, plants allocate resources among three major conflicting functions: reproduction, growth, and maintenance (i.e., metabolism or defense) [2]. In general, natural selection should favor individuals exhibiting higher lifetime reproductive values. However, one reproductive event can have costs, and these are usually quantified as reduced future fecundity and/or survival [3] and reduced growth [4]. This situation can be reflected, for instance, within individual plants. In *Ilex aquifolium*, the branches that produce fruits grow less than non fruiting branches [5], which demonstrates a tradeoff between growth and reproduction.

Dioecious plants (with staminate flowers produced on some plants and pistillate flowers on other plants) provide an excellent opportunity for examining the tradeoffs in resource allocation related to plant reproduction. In these species, resource allocation to reproduction is frequently asymmetrical between individuals of the two sexes [6]. Typically, female plants allocate a greater fraction of their resources (commonly measured as proportional biomass) to reproductive structures than their male counterparts [7], [8], including some of our study species, *Chamaedorea* palms [9], [10]. It is known that, in comparison to female plants, male plants produce more flowers [11], [12], which demand a significant use of nutrients [13], [14]. However, when the full reproductive season is considered, female plants incur in a considerably higher investment in reproduction, largely due to the ripening of fruits and seeds [8], [15]. In female plants of dioecious species deploying greater allocation of resources to reproduction than male plants, a tradeoff between reproduction and growth can occur, negatively impacting growth because part of the resources that could be used for the production of new leaves, stems and roots, are shifted to the production of flowers, fruits and seeds. In female plants investing a higher amount of nutrients (P, N, Mg, Ca) in reproduction than male plants, the decrease in nitrogen levels limits the production of new leaves [15], [16]. Given this predominant tradeoff, female plants are expected to protect their limited growth potential against tissue loss via herbivory. Increased allocation to defense in female plants is crucial, and feasible, particularly if plants produce Carbon-based defensive compounds, a resource that is not so limited, in comparison to other scarcer nutrients [17]. In contrast, male plants have a comparatively higher availability of resources that can be deviated to growth, rather than to defense. Increased growth in male plants does not demand a high investment in defense, as these plants can compensate for tissue lost to herbivory. Differences in resource allocation to deploy resistance and tolerance traits should result in differences in herbivory damage between sexual morphs: male plants should exhibit higher levels of herbivory than female plants [18], [19].

Resource allocation tradeoffs between reproduction and growth in female plants have been documented in a variety of species, including herbaceous plants, shrubs and trees (e.g., *Silene alba*: [20], *Corema conradii*: [15], *Ilex aquifolium*: [13], respectively). Independently of assessing differential reproductive allocation, some studies have shown that male plants of several dioecious species exhibit greater growth than female plants [21]–[23].

In addition to the growth-reproduction tradeoff, there are reasons to expect a tradeoff between growth and defense. Some precursor molecules are needed for both synthesis of proteins and secondary (defensive) metabolites [17]. Thus a tradeoff between growth and defense may be inevitable. Given that herbivory represents an important factor affecting plant fitness, anti-herbivore traits, such as secondary compounds [24], and physical defenses, such as trichomes [25] and toughness [26], [27], have been regarded as adaptive responses of plant species to deal with such challenges (see [28]). Therefore, in dioecious species, patterns of differential resource allocation to reproduction between male and female plants may play an important role in determining the patterns of defense and herbivory in the field.

The tradeoffs between reproduction, growth and defense, and their consequences on herbivory of dioecious plants in the wild, have attracted the attention of researchers for years. Recently, Cornelissen & Stiling [19] performed a comprehensive meta-analysis involving 54 studies and found evidence that, overall, male plants exhibited significantly higher growth, had lower levels of putatively defensive attributes, and sustained more damage by herbivores than female plants. Surprisingly, the authors's meta-analysis did not detect significant differences in reproductive effort between male and female plants, an important premise for the deployment of the expected relationships among growth, defense and herbivory [29]. Furthermore, most studies have assessed tradeoffs between two functions and to our knowledge resource allocation tradeoffs among reproduction, growth and defense, and their herbivory levels, have been investigated only in two species of dioecious plants. In one of them, with *Baccharis halimifolia*, the authors considered only the number of flowers as a measure of allocation to reproduction [30]. This measure may not represent the total allocation to female reproduction, as fruit ripening is not taken into account. In the other study, with *Chamaedorea alternans*, resource allocation to defense was assessed only qualitatively (presence or absence of secondary compounds) [10], [31], [32]. Thus, further research simultaneously assessing tradeoffs among reproduction, defense and growth, and the reflection of that on herbivory should provide both a more integrated view of allocation conflicts and evidence to help us understand the apparent lack of consistency in the reported cases in the literature.

To this end, we compared three sympatric species of dioecious palms (*Chamaedorea* spp.) from a Mexican tropical rain forest. We first assessed differences in biomass allocation to reproduction and then monitored growth for several years, measured defensive traits and estimated herbivory. We made an effort to control for the possible confounding effects of spatial heterogeneity, apparency, and phenology. We used these three sympatric congeneric species because they present detectable variations in herbivory, habitat distribution and abundance—variables that may potentially lead to local divergence in response. In a recent phylogenetic analysis of *Chamaedorea*, including a total of 63 species, the three studied species were placed in different clades [33]. This provides a means to assess if, despite the contrasting ecological conditions in which the species grow, and their phylogenetic positions, the predicted patterns of allocation and their consequences on growth and plant-herbivore interactions are consistent.

The specific questions we asked were: i) Compared to male plants, do female plants exhibit a greater biomass allocation to reproduction? ii) Do differences in allocation translate into differences in growth, defense and herbivory? iii) Are there consistent responses among the three sympatric species despite the fact they exhibit interspecific contrasts in natural levels of herbivory, microhabitat distribution, and abundance?

## Methods

### Study site

This study was conducted at the Estación de Biología Tropical Los Tuxtlas, a field station of the National University of Mexico (UNAM) (18°34′–18°36′ N, 95°04′–95°09′ W, 150 m a.s.l.), in the state of Veracruz, Mexico. The predominant vegetation of the area is tropical rain forest, with trees up to 35 m tall. Understory vegetation is dominated by palms, a crucial component of the structure and diversity of the forest [34], mainly *Astrocaryum mexicanum,* and *Chamaedorea* spp. Mean annual rainfall is 4700 mm and mean monthly temperature is 23.4°C [35]. Details of the natural history and ecology of the study zone can be found in González-Soriano *et al*. [36].

### Study species

The genus *Chamaedorea* includes 107 species, distributed from Mexico to Brazil and Bolivia [37], all of them dioecious. At the study site there are six sympatric species of C*hamaedorea*, including the three selected for this study: *Chamaedorea alternans* H. Wendl., *Chamaedorea pinnatifrons* (Jacq.) Oerst., and *C. ernesti-augusti* H. Wendl. The three species show a gradient in the degree of natural herbivory in the direction *C*. *alternans* > *C. pinnatifrons* > *C. ernesti-augusti*. These species also vary in their abundance, showing the same ranking as herbivore damage. In addition, the species have a distinguishable degree of microhabitat separation, with *C. alternans* and *C. pinnatifrons* distributed in sites of lower elevation, while *C. ernesti-augusti* typically occurs in sites of slightly higher elevation (400 m above sea level) (R. Dirzo, unpub. data). The foliage of the three species is consumed by small Chrysomelid beetles. *C. alternans* is the main food source of larvae and adult *Calypthocephala marginipennis* (Chrysomelidae: Casidinae) [32]; *C. pinnatifrons* is consumed by the larvae of the same species, while *C. erenesti-augustii* shows leaf damage by an unidentified beetle (V. Cepeda-Cornejo, unpub. data).

### Resource allocation to reproduction

In order to assess resource allocation to reproduction, we used available data from the literature for two of the species. Resource allocation to reproduction in terms of proportional biomass allocated to reproductive structures had been measured previously in *C. ernesti-augusti,* by Bullock [9], and in *C. alternans*, by Oyama and Dirzo [10]. Such studies represent an ideal source of information for the present work, as they were carried out with two of our study species, *C. alternans* (formerly known as *C. tepejilote* but now assigned to its correct identity of *C. alternans* [38]), and *C. ernesti-augusti*. Such studies were carried at our study site, and specifically in the general area where we conducted this work. Furthermore, such available data allowed us not to have to destructively sample whole plants from our study populations in the Los Tuxtlas preserve. In the case of *C. pinnatifrons* we assessed biomass allocation to reproduction following the same general methodology as in the other two studies, using plants available in a population adjacent to the preserve. Six male and six female plants of *C. pinnatifrons* were harvested during the peak of the reproductive season of 2002. Plant height ranged from 1.16 m to 2.42 m in female plants and from 1.17 m to 1.74 m in male plants. Plants were dug out, taking care not to damage the roots and each plant was separated into vegetative structures (roots, stem and leaves) and reproductive structures (flowers and inflorescences in the case of male plants, and fruits and infructescences in the case of female plants). All plant materials harvested were kept at 80°C in a drying oven for 48 h, until dry weight was constant, after which they were weighed. With the dry weights of each structure we calculated the proportional allocations in the same way as in the previous studies with the other two species [9], [10].

### Field sampling design

In order to measure growth, leaf toughness and herbivory in the three study species we randomly chose 15 male-female pairs of plants for *C. alternans* and *C. ernesti-augusti*, and 12 pairs for *C. pinnatifrons* in March, 2001. We used a pair-wise sampling protocol to control for spatial heterogeneity. Pairs of plants were chosen to match plant height, as much as possible. Indeed, the initial height was statistically indistinguishable between members of pairs (paired *t*-tests: *C. alternans*, *t*_14_ = 1.07; *P* = 0.30; *C. pinnatifrons*, *t*_11_ = 1.15; *P* = 0.27; *C. ernesti-augusti*, *t*_14_ = 1.08; *P* = 0.30). In addition, the distance between plants of each pair was short, less than 3 m, which reduced possible heterogeneity in soil, light, humidity and local herbivore abundance. These plants were used to measure growth in terms of height increment and leaf production. An additional set of 15 male-female pairs of plants for *C. alternans*, 15 pairs for and *C. ernesti-augusti*, and 14 pairs for *C. pinnatifrons* was established in June, 2005, using the same protocol as in the first group of plants, to measure relative growth rate in terms of estimated above-ground biomass production. Again, the initial height was statistically indistinguishable between the pairs of this second group (paired *t*-tests: *C. alternans*, *t*_14_ = 0.13; *P* = 0.89; *C. pinnatifrons*, *t_13_* = −1.85; *P* = 0.09; *C. ernesti-augusti*, *t_14_* = 0.087; *P* = 0.44).

**Figure 1:**
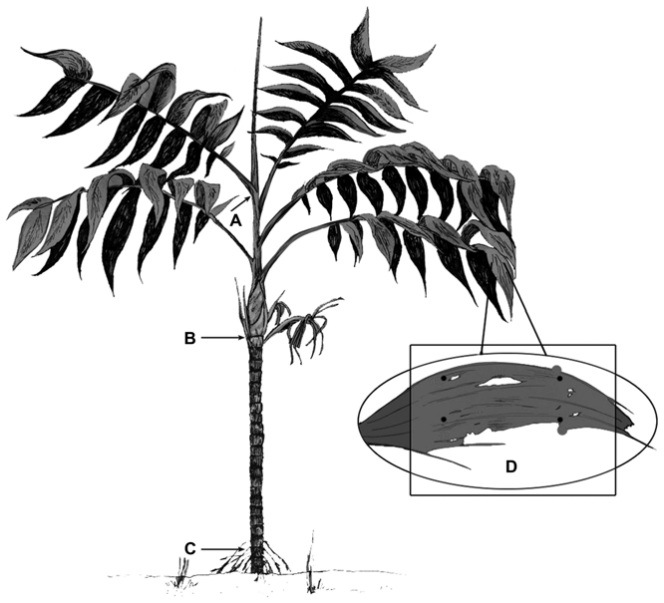
Schematic representation of *Chamaedorea alternans*. Distance between points A and C represents total height. Distance between A and B represents stem height. D indicates demarcated area (encompassed by the four dots) to measure herbivory rate.

For each plant we measured initial height, basal diameter, number of leaves and, for the plants selected in June, 2005, length of rachis of each one of the leaves. Initial height was measured as the distance between the basal diameter (Figure 1C) and the point of departure of the newest leaf from the apical meristem (Figure 1A). Initial basal diameter was measured with a digital caliper to the nearest 0.01 mm. Leaf production rate was measured as the number of new leaves produced, as a function of the initial number of leaves. To monitor growth and calculate growth metrics (see below), the initial leaves and the points of initial measurement of basal diameter were marked with permanent, water proof ink.

### Growth metrics

We used several metrics to describe growth of male and female plants.

Total height increment (THI) was calculated from 2001 to 2002, using the increment in stem height (see Figure 1A-C), as THI  =  (ln H_final_ – ln H_initial_)/t, where H  =  height and t  =  time in years [39].

Leaf production rate (LPR) was calculated both from 2001 to 2002 and again from 2005 to 2007 as: LPR =  newly produced leaves/(initial number of leaves/t), where t  =  time in years.

Relative growth rate (RGR) was assessed as the change in aboveground biomass (stems and leaves) from the initial time (2005) to the final time (2007). We first measured stem length from the basal diameter (Figure 1A) to the distal point of the most recently produced metamer (Figure 1B); therefore, this variable considers the addition of new metamers and the elongation of internodes. RGR was calculated using non-destructive estimates of plant biomass through allometric equations (see below) as RGR  =  (ln W_final_ – ln W_initial_)/t, where W  =  biomass in dry weight and t  =  time in years [39]. Since this metric does not take into account leaves that fall during the interval, we estimated leaf mass based on all the produced leaves from each plant during the two years of study.

Net biomass production (BP) in both stem and leaves was calculated from 2005 to 2007 as: BP  =  above-ground biomass produced/(initial above-ground biomass/t) where t  =  time in years. Plant biomass was calculated by allometry, calculating regression equations for stems and leaves (in dry weight). Given that the development of these equations required destructive harvesting we deemed necessary to use plants independent from our matched pairs to avoid damage in these plants used for long-term monitoring. We developed regression equations by harvesting plants of different sizes, of all three species, located outside the reserve, and measuring lengths and diameters of stems, and lengths of rachis of leaves of plants of different sizes (see [40]). We dried these materials at 60°C in an oven for at least 96 h (until constant weight) and measured dry weight of these structures. Data were fitted to regression equations (linear for stem, and quadratic for leaves), using JMP, vers. 6.0. Best fitting was assessed using the highest coefficient of determination (R^2^). The resulting equations, which were used to estimate biomass (weight in g) from standing measurements of height (cm), diameter (mm), and rachis length of leaf (cm) are shown in Table 1 (only the best fitting equations are shown).

**Table 1:** Regression models used for estimates of above-ground biomass.

Species		*b*_0_	*b*_1_	*b*_2_ *X* ^2^	df	*F*	*R* ^2^
*C. alternans*	Leaf	−11.158	0.252	0.0006×(*X* -91.164)^2^)	1, 30	546.5***	0.94
	Stem	5.792	0.037		1, 70	2077.9***	0.96
*C. pinnatifrons*	Leaf	−2.693	0.115	0.0019×(*X* -40.161)^2^)	1,42	140.6***	0.87
	Stem	−0.508	0.038		1, 53	3104.5***	0.98
*C. ernesti-augusti*	Leaf	2.483	0.164	0.0018×(*X* -29.882)^2^)	1, 39	184.18***	0.96
	Stem	−1.530	0.041		1, 47	14472.8***	0.95

Models for biomass prediction are of the form Y  =  *b*_2_ *X* ^2^ + *b*_1_ *X* + *b*_0_ in leaves and Y  =  *b*_1_ *X* + *b*_0_ in stems. Y is the dependent variable (dry weight in g), *X* is the independent variable (leaves: rachis length [cm], stem: height [cm] × diameter [mm]), *b*_0_, *b*_1_ and *b*_2_ are constants in the equation. *** P<0.0001

### Defense

In the three study species, we used two independent variables to estimate defense, leaf toughness [24] and secondary metabolites. In particular, for the latter, we quantified total phenolic compounds concentration, as it is known that these defensive compounds are present in the study species [32] and because theory predicts that the study species, being shade tolerant, slow growth species, are likely to use Carbon-based defenses of high molecular weight [41], [42]. To measure toughness we randomly selected two leaflets from leaf # 2 on the phyllotaxis in each plant of the 15 selected (male-female) pairs in 2001. We took three measures for each leaflet in different points (basal, middle and apical) using a 500 g leaf penetrometer (Chatillon, Gauge-R CATL 516–500).

To measure total phenolics concentration, we collected leaves from a selected group of 17–22 plants (different from the pairs used for growth, to prevent alterations of the growth of such paired plants) of each sex, of each species. Two randomly selected leaflets were collected from leaves in positions #2 and #3 in the plant's phyllotaxis. This material was kept in liquid nitrogen and transported to the laboratory, where a fraction (1 g) was macerated with 50 ml of liquid nitrogen in a mortar. The extraction of the phenolic compounds was made in 80% aqueous methanol for 24 h, and the Price and Butler method, modified by Graham, was used to assess total phenolics [43]. The Prussian blue reaction was measured at 720 nm in a spectrophotometer (Genesis 20), and was compared to a standard curve obtained from tannic acid.

### Herbivory

An initial estimation of herbivory was obtained by calculating standing levels of damage in the 15 pairs of plants of each species selected in 2001. To this effect, for each plant we collected four randomly selected leaflets from the 3^rd^ leaf in the plant's phyllotaxis. The leaflets were pressed and dried. Digital images were taken and analyzed using the software WinDias ver. 2.0 (Delta-T Devices) to calculate percentage leaf area damaged. WinDias software measures the actual (remaining) leaf area (ALA), and estimates the potential leaf area (PLA) (undamaged) by drawing out the contours and filling in the spaces of damaged areas. When damage was very extensive we estimated PLA by matching the remaining area with a comparable intact leaflet of the same species. Leaf herbivory (H), defined as the percentage of leaf area damaged, was calculated as: H  =  (PLA-ALA/PLA)*100. The H average of all leaves sampled from each plant was used to estimate herbivory per plant.

In addition, we complemented such standing measures of damage by measuring the rates of herbivory, using the damage accumulated in 286 days (September 30, 2001-July 13, 2002) on plants from the 15 pairs. Four intact leaflets from a leaf at position #2 in the phyllotaxis were randomly selected. A section of the leaflet was marked out by four indelible-ink points painted on the underside of the leaflet, to demarcate a rectangle of 3×18 cm in *C. alternans*, 2.6×9 cm in *C. pinnatifrons*, and a square 10×10 cm in *C. ernesti-augusti*. Onto this demarcated area, we placed a transparent grid (2 mm squares) to count the number of intact squares and squares with herbivory and the ratio of squares with damage:total squares was used to measure leaf damage. After 286 days we measured the percentage of leaf area damaged and calculated the rates of herbivory (HR) as:

HR  =  (% damaged area _final_ - % damaged area _initial_)/t, where t =  time in days.

### Statistical analysis

Intersexual differences in resource allocation to reproduction were compared with a Mann-Whitney U test for independent samples (Statistica, 1984–2000 by StatSoft, Inc.), and the possible variation in allocation of biomass to reproduction (percentage of total biomass of inflorescences or infrutescences) as a function of plant height (see [10]) was examined by linear regression (JMP vers. 6.0) in *C. pinnatifrons*. Growth variables were analyzed using nested multivariate analyses of variance (MANOVA), one for each of the two periods and their corresponding variables, as indicated above. MANOVAs were followed by nested ANOVAs. The ANOVA model included the effect of sex and species, and plant pair nested within species as a random-effect, as well as the sex × species interaction. This was applicable to all variables except total phenolic concentration (given that these were plants independent to the 15 pairs). In those cases in which the interaction term was significant, indicating that intersexual differences varied across species, we perfomed a post-hoc analysis, using the test slices comparison (JMP vers. 6.0), which analyzes each of the levels of the interaction. Given that the studentized residuals of all variables, except leaf production, were not normally distributed (after testing with a Shapiro-Wilks test (JMP vers. 6), we performed a Box Cox transformation (JMP vers. 6), which yielded normalized data. In addition to nested ANOVAs, differences among species in herbivory rates were analyzed with a Tuckey test.

## Results

### Resource allocation to reproduction

**Figure 2:**
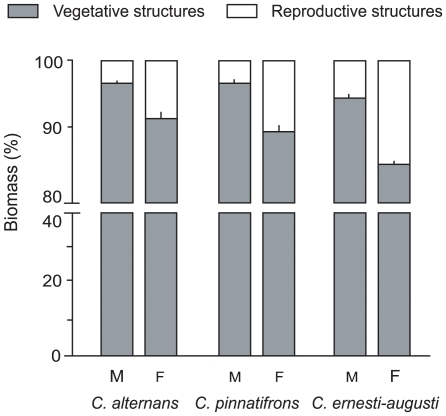
Biomass allocation to reproductive and vegetative structures in male (M) and female (F) plants of the three studied species. Data for *C. alternans* and *C. ernesti-augusti* were obtained from Oyama and Dirzo (1988), and Bullock (1984), respectively. Error bars denote standard error.

Resource allocation to reproduction in *C. pinnatifrons* was 3.04 times higher in female plants, compared to male plants (Figure 2). This difference was highly significant (*U_0.05(2),_*_6,6_ = 0.00, *P* = 0.0039). This result is consistent with what is known for the other two species of study (Figure 2): in *C. alternans* resource allocation to reproduction in female plants was 2.55 higher than in male plants [10], and 2.75 for *C. ernesti-augusti* [9]. Biomass allocation to reproductive structures (i.e., the percentage of total biomass) did not change with plant height (both male and female plants: *F*_1,10_ = 0.2550, *P* = 0.6257).

### Growth metrics

The MANOVA for the growth variables measured in both periods (2001–2002, 2005–2007) was highly significant (*F*_88,76_ = 2.38, *P*<0.0001 and *F*_135,114_ = 2.5, *P*<0.0001, respectively). Male and female plants differed in growth in both years of the study (*F*_2,38_ = 12.45, *P*<0.0001 and *F*_3,38_ = 2.9, *P* = 0.047, respectively). The species of palms also differed in growth in the two periods (*F*_4,76_ = 13.77, *P*<0.0001 and *F*_6,76_ = 23.04, *P*<0.0001, respectively), and the patterns between the sexes were consistent in all species (interaction sex × species term in the period 2001–2002 [*F*_4,76_ = 1.37, *P* = 0.252], and in the period 2005–2007 [*F*_6,76_ = 1.96, *P* = 0.08]).

**Figure 3:**
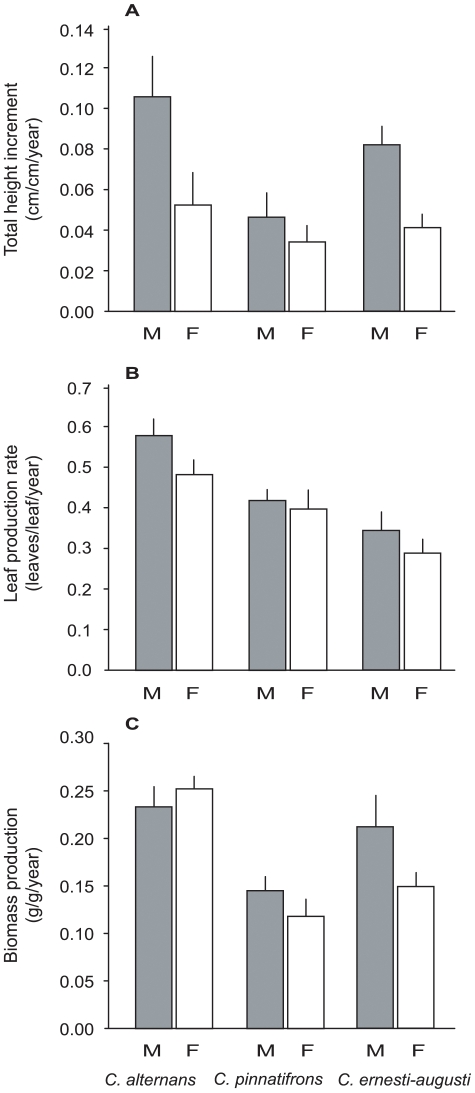
Comparison between male (M) and female (F) plants of four growth variables of the three studied species. A) total height increment, B) leaf production rate, C) above-ground biomass production. Error bars denote standard error.

The subsequent ANOVAs on total height increment (THI) and leaf production rate (LPR) (2001–2002, 2005–2007) showed that males grew faster than females (Table 2, Figure 3A, B). In *C. alternans*, growth expressed in terms of THI was higher in male plants compared to female plants (Figure 3A), with a two-fold difference in stem elongation (14.47±2.37 cm/yr vs. 6.95±2.09 cm/yr, respectively). A similar response was found in *C. pinnatifrons*, with a 31% higher increment in male than in female plants (7.61±1.96 cm/yr vs. 5.2±1.02 cm/yr) and *C. ernesti-augusti*, with a 46% difference in male vs. female plants (8.69±0.75 cm/yr vs. 4.64±0.68 cm/yr, respectively).

**Table 2:** Nested ANOVAs to assess the effects of species, sex and their interaction, on four growth variables measured in two periods.

	*2001*–*2002*			*2005*–*2007*			
	*THI*	*LPR*		*RGR*	*BP*	*LPR*	
Source of variation	*F*	*F*	df	*F*	*F*	*F*	df
Species	3.08*	17.89***	2	2.16	17.96***	10.30***	2
Sex	20.30***	5.26*	1	2.15	5.75*	4.23*	1
Pair[Species]	0.73	0.47	39	0.76	0.83	0.54	41
Species × Sex	2.19	0.71	2	0.10	4.26*	1.65	2

Total height increment (THI) and leaf production rate (LPR) for 2001–2002; relative growth rate (RGR), net biomass production (BP) and LPR for 2005–2007. *P<0.05, *** *P*<0.0001.

In the case of LPR in 2001–2002, male plants produced significantly more leaves than female plants (Table 2, Figure 3B). In *C. alternans* LPR was 1.36-times higher in male than female plants (2.83±0.17 leaves/leaf/yr vs. 2.08±0.17 leaves/leaf/yr, respectively). *Chamaedorea pinnatifrons* had a slightly higher increment in male than in female plants (2.4±0.2 leaves/leaf/yr vs. 2.2±0.22 leaves/leaf/yr, respectively) and male plants of *C. ernesti-augusti* produced 1.48-times more leaves than female plants (3.25±0.16 leaves/leaf/yr vs. 2.19±0.2 leaves/leaf/yr, respectively). Given that in 2005–2007 we measured leaf production in order to estimate biomass production, we were able to calculate, again, LPR and check for consistency of results with the data from the first period. Again, the intersexual differences were consistent across species, with male plants significantly producing more leaves than female plants (Table 2). In the period 2005 to 2007 LPR in *C. alternans* was 1.25-times higher in male than in female plants (2.99±0.18 leaves/leaf/yr vs. 2.37±0.17 leaves/leaf/yr, respectively); in *C. pinnatifrons* it was 1.21-times higher in male than in female plants (2.86±0.12 leaves/leaf/yr vs. 2.2±0.1 leaves/leaf/yr, respectively) and *C. ernesti-augusti* exhibited 1.21-times more LPR in male than female plants (2.58±0.17 leaves/leaf/yr vs. 2.13±0.17 leaves/leaf/yr, respectively).

A comparison of RGR in the period 2005–2007 did not detect differences across species and plant sex (Table 2). However, net biomass production (BP) at the end of the period showed intersexual differences depending on the species. Male plants of *C. ernesti-augusti* produced 30% more net biomass than female plants (*F*_1,41_ = 6.18, *P* = 0.017) and in *C. pinnatifrons* male plants produced 19% more biomass (*F*_1,41_ = 6.85, *P* = 0.012), while no significant differences were detected in the case of *C. alternans* (*F*_1,41_ = 0.99, *P* = 0.325) (Table 2, Figure 3C).

### Defense

**Figure 4:**
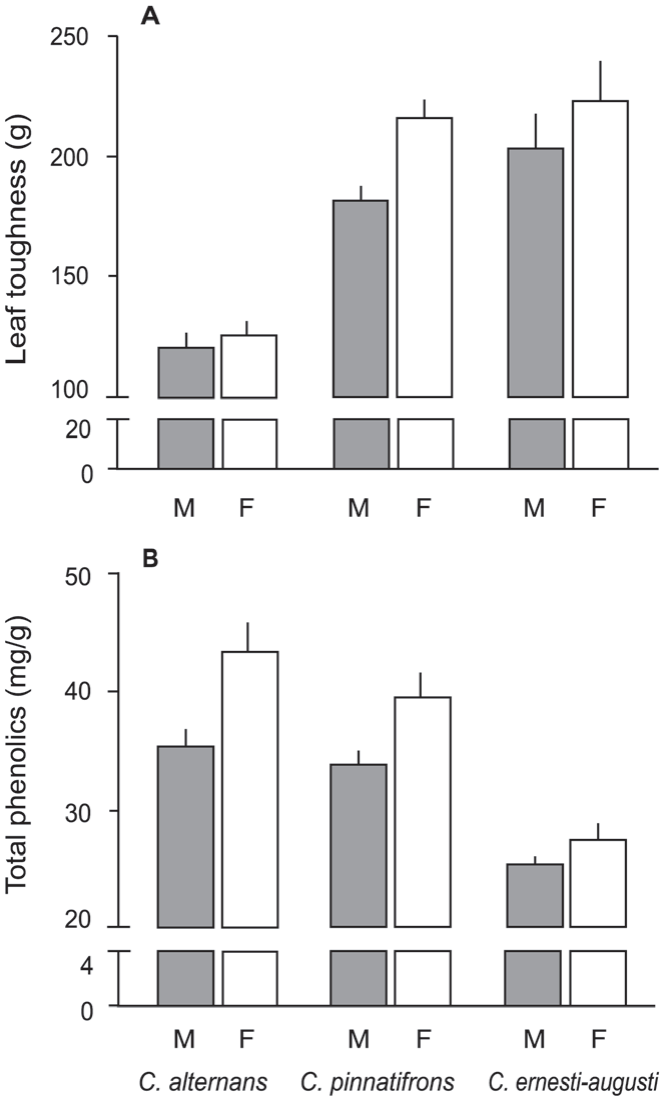
Comparison between male (M) and female (F) plants in two defensive characteristics of the three studied species. A) leaf toughness, B) total phenolic concentration. Error bars denote standard error.

ANOVA detected differences between species in both leaf toughness and total phenolics (Table 3). Toughness values were higher in female plants than in male plants in all three species (a 4% difference in *C. alternans*, 9% in *C. ernesti-augusti,* and 16% in *C. pinnatifrons*) (Table 3, Figure 4A). Similarly, total phenolics concentration was consistently higher in female than in male plants, with contrasts that ranged from 18% (*C. alternans*), to 14.5% (*C. pinnatifrons*), to 7.6% (*C. ernesti-augusti*) (Figure 4B).

**Table 3:** Nested ANOVAs to assess the effects of species, sex and their interaction, on two defensive characteristics and two estimates of herbivory.

	Defensive characteristics				Herbivory			
	*Leaf toughness*		*Total phenolics*		*Standing damage*		*Herbivory rate*	
Source of variation	*F*	df	*F*	df	*F*	df	*F*	df
Species	42.83***	2	52.47***	2	45.47***	2	72.84***	2
Sex	6.51*	1	12.64***	1	24.78***	1	63.80***	1
Pair[Species]	0.79	39			0.58	37	1.24	39
Species × Sex	0.65	2	0.91	2	1.22	2	2.0948	2

* P<0.05, **P<0.001, *** P<0.0001.

### Herbivory

**Figure 5:**
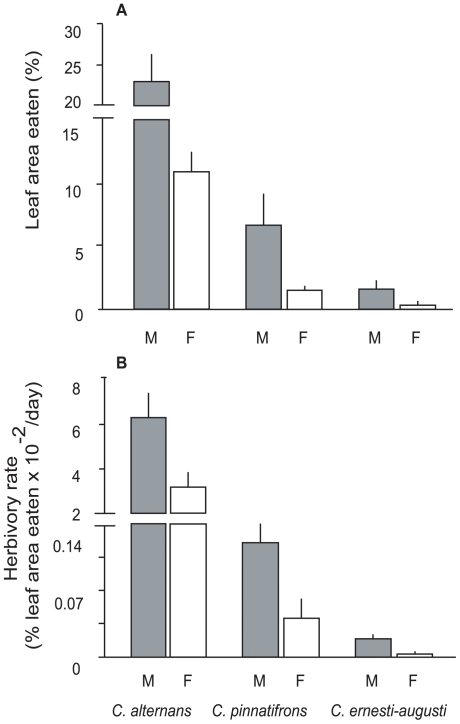
Comparison of two measures of herbivory between male (M) and female (F) plants of the three studied species. A) standing levels of damage measured in 2002, B) rates of herbivory measured in 2001–2002. Error bars denote standard error.

Intersexual variation in natural herbivory was also highly significant (Table 3, Figure 5) and consistent across the three species. In all three species standing damage in male plants was 2–4.7 higher in male than female plants (Figure 5A). Rates of herbivory showed the same pattern, with differences that were 1.98, 3.85, and 7.50 times higher in male than in female plants of *C. alternans*, *C. pinnatifrons* and *C. ernesti-augusti*, respectively (Figure 5B). In addition to the intersexual variation, overall standing damage showed a gradient across species in the direction *C. alternans* > *C.pinnatifrons* > *C. ernesti-augusti* (*Q* = 2.44, alpha = 0.05) (Figure 5A). Herbivory rates showed the same gradient (*Q* = 2.43, alpha = 0.05) (Figure 5B).

## Discussion

The results of this study show an asymmetric allocation of biomass to reproduction between female and male plants, where the biomass allocated in female plants was higher than in male plants. Correlated with this, the growth observed in stem, number of leaves and biomass production was greater in male than female plants of all three species. In addition, female plants were significantly better defended (leaf toughness and phenolic compounds) than male plants, and herbivore damage was greater in male than in female plants. These results support the predictions of the consequences of intersexual differences in resource allocation on reproduction, growth and defense, and its repercussions on herbivory in dioecious plant species. To our knowledge, this is the first study that integrates the relevant elements for the Resource Allocation Principle (differential resource allocation to reproduction, growth and defense) and its consequences for herbivory, in a single system, in this case a group of long-lived, tropical sympatric species of the same genus. In addition, it is worth noting that out of 54 studies available in the literature [19], only a minor fraction (3) analyzed species from tropical forests, and only one was done involving palms. This is critical, as it is known that the proportion of dioecious species at the global scale is particularly high in the tropics [44] and that palms are a crucial structural component of tropical forests [34].

Our study is largely based on a design of replicated pairs of neighboring male and female plants followed in multi-year observations. This is relevant in that such design minimizes the effect of uncontrolled variables for the comparisons between male and female plants. Indeed, it is possible that one of the reasons that may be responsible for the inconsistency of intersexual patterns reported in the literature [19] may be the lack of control in microhabitat differences among plants. Although most of the plant variables in this study were based on our matched-pair design, some variables had to be measured from independent plants, as this required destructive sampling (biomass allocation to reproduction, allometric relationships and estimation of phenolic compounds). We posit that this might not be a significant limitation, as those plants in which destructive sampling was necessary were located in the same general area and belong to the same populations of the matched pairs. This however is a possible caveat that needs to be taken into account in the following discussion of our results.

### Intersexual differences in resource allocation to reproduction

The difference in biomass proportions allocated to reproduction between sexual morphs of our study plants (∼3-times greater in female plants) falls within the range found for other dioecious species: 1.7 in *Corema conradii* [15], 2.5 in *Salix sachalinenesis* [45], 3.3 in *Xanthoxylum americanum* [46] and 4.4 in *Eurya japonica* [8]. Consistent with this, intersexual contrasts in nutrient content have also been documented [14]. In *Salix glauca* the concentration of P and N in seeds is 2.5 and 1.9 times higher than that of pollen [16]. Fruit formation in *Corema conradii* demands 1.6 times more Mg and 2.1 times more Ca than male flowers production [15]. In contrast, a few studies have failed to detect differences in reproductive effort in terms of biomass [47] or number of flowers [48]. In fact, surprisingly, Cornelissen & Stiling's [19] meta-analysis failed to detect an overall intersexual difference in terms of reproductive biomass. Such inconsistency of results might be explained by the timing and type of reproductive structure that is analyzed. In some studies, for example, allocation to reproduction has been considered in terms of flowers. Although reproductive allocation in terms of biomass and nutrients during the flowering stage may be greater for male plants [11], [13], [14], female plants undergo a greater reproductive effort when the entire reproductive process is considered, including the flowering, fruiting and seed dispersal periods [8] and so male-female comparisons of allocation to reproduction need to take all these stages into consideration. Other reasons why differences in reproductive allocation are smaller than expected, or even absent, may be “the currency” used to estimate allocation (carbon or nutrients) and methods of estimation of reproductive allocation (biomass, respiration or photosynthesis). Environmental heterogeneity may also lead to seemingly inconsistent results. For example, Reekie and Bazzaz [49], found that variation in reproductive effort (proportion of resources diverted to reproductive organs) depends of availability of light and/or nutrients, thus emphasizing the need for controlling environmental variation to compare intersexual allocation to reproduction.

### Intersexual differences in growth

Most of the expectations regarding a resource allocation tradeoff between reproduction and growth were met by the three sympatric plants of this study. Female plants of the three species of *Chamaedorea* exhibited a poorer performance in essentially all growth variables we measured. In general, out of twelve possible intersexual comparisons (THI, LPR, RGR and BP in all three species), eight showed significantly higher values in male than in female plants, and four were statistically non-significant. Furthermore, the intersexual differences in leaf production rates are consistent with those found in a previous study with *C. alternans* in the same study site although, surprisingly, that study did not detect significant contrasts in height increment [31].

### Intersexual differences and plant-herbivore interactions

The amount of resources that a plant allocates to reproduction has been found to have significant consequences for growth and this in turn affects a host of additional variables relevant for plant performance in the field, including their interactions with herbivores. Associated to differences in growth, female plants are expected to increase the allocation to defenses for the protection of vegetative tissue (e.g., leaves), because slower growth rates make it more difficult for a plant to replace damaged tissue [50]. Male plants, in contrast, could deploy greater growth and faster leaf turnover rates, sould have greater capacity to compensate for tissue loss to herbivory, and would be under lesser selection for allocation to defenses. Accordingly, we found evidence that resource allocation to defense is greater, and growth is lower in female than in male plants. This is compatible with an argument of a growth-defense tradeoff [17]. Particularly, female plants in understory tropical forests can produce defensive compounds based on Carbon, a resource that is not so limited, in comparison to other scarcer nutrients (e.g. nitrogen) [17]. In accordance with this, we found that female plants had higher concentrations of total phenolic compounds. Likewise, leaf toughness, a physical trait known to be correlated with tannin concentration [51], and known to reduce herbivory [26], [27], was also significantly higher in the foliage of female plants of all three study species. Several studies support the expectation of greater allocation to Carbon-based defenses in the leaves of female plants, as compared to male plants of dioecious species, including the trees *Acer negundo* [22], and *Salix rigida* [21].

As a result of intersexual differences in defense, herbivory levels are expected to be higher in male than female plants and, indeed, differences in herbivory have been associated to contrasts in leaf palatability as determined by the plant's secondary chemistry (see [18], [22], [26], [52], [53]). Leaves with low concentration of secondary metabolites and less toughness could explain that male plants had higher herbivory than female plants in our three study species. In their meta-analysis, Cornelissen and Stiling [19] found that the available literature supports such a trend for male-biased herbivory in dioecious plants in general.

The differences in growth rates and both physical (toughness) and chemical (phenolic compound) defenses between sexes suggest alternative anti-herbivory mechanisms (for male and female plants) to deal with herbivory: greater investment in defenses, or resistance (female plants); and compensatory growth, or tolerance (male plants). Resistance is any plant trait that reduces the preference or performance of herbivores (e.g. thorns, hairs, unpalatable secondary chemicals) [54]. Tolerance is the ability of plants to regrow and/or reproduce after herbivory. Some of the mechanisms involved in increased tolerance are high relative growth rates, increased branching or tillering, increased leaf photosynthetic rate and increased percentage of fruit set [50], [54]. This alternative defense argument is compatible with our findings: male plants seem to deploy tolerance (or at least a lower degree of resistance) while female plants deploy resistance (or a lower degree of tolerance) to deal with herbivores in their natural environment.

### Perspectives

In this paper we analyzed both the reproduction-growth and growth-defense tradeoffs. However, other tradeoffs are possible, for instance: current reproduction-survival, current reproduction-future reproduction, and reproduction-defense [3], [55]. It is foreseeable that these additional tradeoffs may influence the tradeoffs we studied. Further work is warranted in which the reproduction-growth and growth-defense tradeoffs are analyzed in the context of other relevant tradeoffs depending on the species of study.

Although our results are based on a study of three sympatric and congeneric species, the consistency of results is striking, given that the species exhibit differences in microhabitat distribution and in the natural levels of herbivory (cf. Study species and Results sections). Furthermore, a recent phylogenetic analysis of *Chamaedorea* [33], shows that the three studied species are located in different clades, suggesting that similarities in growth, herbivory and defense among species could be the result of convergent evolution in response to similar selection pressures (i.e., herbivory) driven by asymmetrical allocation to reproduction. This is an aspect that warrants further research.
